# Increased Levels of sCD30 Have No Impact on the Incidence of Early ABMR and Long-Term Outcome in Intermediate-Risk Renal Transplant Patients With Preformed DSA

**DOI:** 10.3389/fmed.2021.778864

**Published:** 2021-11-08

**Authors:** Thomas Drasch, Christian Bach, Markus Luber, Bernd Spriewald, Kirsten Utpatel, Maike Büttner-Herold, Bernhard Banas, Daniel Zecher

**Affiliations:** ^1^Department of Nephrology, University Hospital Regensburg, Regensburg, Germany; ^2^Department of Internal Medicine 5-Hematology and Oncology, University Hospital Erlangen, Friedrich-Alexander University Erlangen-Nürnberg, Erlangen, Germany; ^3^Department of Internal Medicine 3-Rheumatology and Immunology, University Hospital Erlangen, Friedrich-Alexander University Erlangen-Nürnberg, Erlangen, Germany; ^4^Institute of Pathology, Regensburg University, Regensburg, Germany; ^5^Department of Nephropathology, University Hospital Erlangen, Erlangen, Germany

**Keywords:** kidney transplantation, donor-specific anti HLA antibodies, sCD30, risk stratification, ABMR, antibody-mediated rejection

## Abstract

**Background:** It is still incompletely understood why some patients with preformed donor-specific anti-HLA antibodies (DSA) have reduced kidney allograft survival secondary to antibody-mediated rejection (ABMR), whereas many DSA-positive patients have favorable long-term outcomes. Elevated levels of soluble CD30 (sCD30) have emerged as a promising biomarker indicating deleterious T-cell help in conjunction with DSA in immunologically high-risk patients. We hypothesized that this would also be true in intermediate-risk patients.

**Methods:** We retrospectively analyzed pre-transplant sera from 287 CDC-crossmatch negative patients treated with basiliximab induction and tacrolimus-based maintenance therapy for the presence of DSA and sCD30. The incidence of ABMR according to the Banff 2019 classification and death-censored allograft survival were determined.

**Results:** During a median follow-up of 7.4 years, allograft survival was significantly lower in DSA-positive as compared to DSA-negative patients (*p* < 0.001). In DSA-positive patients, most pronounced in those with strong DSA (MFI > 5,000), increased levels of sCD30 were associated with accelerated graft loss compared to patients with low sCD30 (3-year allograft survival 75 vs. 95%). Long-term survival, however, was comparable in DSA-positive patients irrespective of sCD30 status. Likewise, the incidence of early ABMR and lesion score characteristics were comparable between sCD30-positive and sCD30-negative patients with DSA. Finally, increased sCD30 levels were not predictive for early persistence of DSA.

**Conclusion:** Preformed DSA are associated with an increased risk for ABMR and long-term graft loss independent of sCD30 levels in intermediate-risk kidney transplant patients.

## Introduction

Antibody-mediated rejection (ABMR) caused by donor specific anti-HLA IgG antibodies (DSA) is responsible for the majority of graft losses after kidney transplantation and still remains one of the major challenges in transplant nephrology (1). Introduction of the single antigen bead (SAB) assays using Luminex technology has improved both sensitivity and specificity of detecting preformed DSA considerably but has left clinicians with the conundrum that many DSA-positive patients have favorable long-term outcomes.

Attempts have therefore been undertaken to improve the predictive value of the SAB assay. Analysis of immunoglobulin isotypes (2), subclasses (3, 4) or the capacity of the anti-HLA antibodies to bind and activate complement (5–7) have yielded mixed results.

CD30 is a 120 kD glycoprotein and part of the tumor necrosis factor (TNF) superfamily. Besides its constitutional expression on a variety of lymphoid neoplasms, most notably Hodgkin's lymphoma cells, it is expressed on activated T and B cells (8, 9). CD30 signaling via its receptor CD30 ligand (CD153) has been shown to play an important role in the generation of both memory CD8+ T cells and in regulating CD4+ T cell-mediated graft vs. host disease in animal studies (10). Cleavage of membrane-bound CD30 by metalloproteases generates the 85 kD protein soluble CD30 (sCD30). Although the exact biological function of sCD30 remains to be elucidated (11), elevated serum concentrations of sCD30 have been found to correlate with disease activity in patients with systemic lupus erythematosus, granulomatosis with polyangiitis and rheumatoid arthritis [reviewed in (8)]. In 2002, Pelzl et al. first reported increased pre-transplant sCD30 levels to be associated with reduced kidney allograft survival (12). Several following studies confirmed an association of elevated pre- and posttransplant levels sCD30 with rejection episodes or impaired allograft survival (13, 14), whereas other studies could not reproduce these findings (15, 16). Recently, Süsal et al. combined the T cell activation marker soluble CD30 (sCD30) and the SAB assay for risk stratification in two retrospective cohorts of sensitized kidney transplant patients. Remarkably, patients only exhibited an increased risk for graft loss in the presence of both elevated levels of sCD30 and DSA, whereas DSA-positive patients had comparable outcomes to DSA-negative patients in the absence of high sCD30 levels (11, 17, 18). These findings resulted in the hypothesis that DSA can only exert their detrimental effects in patients with a pre-activated cellular immunity as indicated by elevated pre-transplant levels of sCD30.

Of note, the first cohort consisted of 80 highly-sensitized patients all with complement-dependent cytotoxicity panel-reactive antibodies (CDC-PRA) above 85%, 20% of whom were CDC-crossmatch (CDC-CM) positive prior to an intensive desensitization regimen including plasmapheresis and rituximab (17). The second cohort consisted of 385 at least moderately sensitized patients as indicated by either CDC-PRA positivity or ELISA-reactive anti-HLA antibodies. Induction treatment was variable with 11% receiving T-cell depletion and 53% receiving no induction regimen at all. Data on ABMR were not reported (11, 18).

Given the high immunological risk of the hitherto reported cohorts and their variable induction regimens, we asked whether a combination of preformed DSA and elevated sCD30 levels would also be predictive of early ABMR and accelerated graft loss in a homogenous group of intermediate-risk kidney transplant patients all treated with the same non-depleting induction regimen and tacrolimus-based maintenance immunosuppression. These patients had been transplanted prior to the clinical use of the SAB assay and pre-transplant risk stratification was solely based on a negative CDC-CM.

## Patients and Methods

### Study Population

From all patients that received a living or deceased kidney transplant at our institution between January 2005 and December 2015 (*n* = 686), we retrospectively selected all those treated with an anti-IL2-receptor-based induction therapy (basiliximab, Simulect^®^, Roche, Basel, Switzerland) followed by a maintenance regimen consisting of a calcineurin-inhibitor, mycophenolate-mofetil and prednisolone (*n* = 287, Supplementary Table 1). Patients that simultaneously received multiple organs or had received an organ other than a kidney previously were excluded, as were ABO-incompatible living donor kidney transplantations. Kidney-only recipients treated without any induction therapy, depleting-antibody induction, i.e., anti-thymocyte globulins (ATG), or an mTOR-inhibitor-based maintenance regimen, were excluded as well as patients for whom no serum sample was available prior to transplantation (*n* = 8). During the study period, all recipients of a living donor transplant received basiliximab induction. For deceased donor transplantations, induction therapy was determined on an individual basis with no predefined criteria. All patients were transplanted with a negative CDC-CM using current sera. Donor and recipient characteristics as well as clinical data were obtained by careful chart review or were extracted from the Eurotransplant Network Information System (K_X_008). All retrospective analyses were performed with approval of the local Institutional Review Board.

### Detection and Definition of DSA and Donor HLA Typing

Sera taken at the time of kidney transplantation were retrospectively screened for the presence of anti-HLA class I and class II IgG antibodies. Sera from patients with preformed DSA were additionally screened for the presence of DSA at day 14 post-transplantation. All sera were stored at −80°C and heat inactivated at 52°C for 20 min prior to analysis. Screening was done using a commercial solid-phase microsphere-based assay (LSM12, One Lambda Inc., Los Angeles, CA, USA). Sera were analyzed on a LABScan 100 Luminex^®^ (Luminex Corp., Austin, TX, USA) flow analyzer, applying a threshold ratio for positive results of 2.5. In positive sera, HLA specificity was determined by a single antigen assay for HLA class I and / or HLA class II antigens (LABScreen^®^ Single Antigen, Class I or II, respectively, both One Lambda Inc.). The tests were performed according to the manufacturers' instructions, applying a baseline-adjusted MFI cut-off for positive reactions of 1,000. Donor-specificity of anti-HLA antibodies was defined based on the available donor HLA typing data. Donor HLA-typing was performed according to standard Eurotransplant protocols. Typing for HLA-A, B and DR was done for all donors. HLA Cw and DQ typing data were available for 95 (32.2%) and 275 (93.2%) donors, respectively. DP typing was not routinely performed and therefore, anti-DP HLA-antibodies were not evaluated for donor-specificity. If donor-specificity of anti-HLA antibodies could not be determined due to lack of high resolution typing of a donor, they were classified as non-DSA. This occurred in five recipients for HLA class I and in 14 patients for HLA class II antibodies, respectively. However, lack of high resolution typing in the corresponding donors resulted in no potential misclassification with respect to pre-transplant DSA status (yes/no). In case Luminex analysis revealed the presence of antibodies for all different splits of an HLA antigen, the bead with the highest MFI was used for MFI categorization. To categorize patients into DSA positive or negative, both a lower MFI threshold of 1,000 and 5,000 were applied as previously published (11, 19). In patients with more than one DSA, the one with the highest MFI (MFI^max^) was used for categorization.

### Measurement of SCD30

Pre-transplant sera were tested for sCD30 using the ELISA kit of eBioscience (San Diego, USA). Based on previous results, a value of 80 ng/ml was used as the most suitable cut-off for sCD30 testing (18).

### Diagnosis and Treatment of Rejection

All rejection episodes were biopsy-proven. Biopsies were obtained either as protocol biopsies on days 14 and 90 post-transplantation or when clinically indicated. At the time of biopsy, specimens were evaluated according to the most recent Banff classification. For the current study, biopsies from DSA-positive patients were re-evaluated by an experienced nephropathologist (MB-H). Immunohistochemical staining for C4d was complemented when no C4d staining was performed at the time of biopsy and all biopsies were re-classified according to the BANFF 2019 classification (20). Subclinical borderline rejections were not treated. Both clinical and subclinical TCMR were treated with steroid pulses. In case of vascular or steroid-resistant TCMR, anti-thymocyte globulins were given. Any combination of steroids with plasmapherese, intravenous immunoglobulins and/or rituximab was considered adequate therapy for ABMR.

### Statistical Analysis

Statistical analysis was performed using IBM SPSS version 26.0 (SPSS Inc., Chicago, IL, USA). Survival analyses were performed by the Kaplan-Meier method and differences between groups compared using the log-rank test. Differences in baseline characteristics were analyzed by using the chi-square test (Fisher's exact when appropriate), Mann-Whitney U- or the Kruskall Wallis test. A *p* < 0.05 was considered statistically significant.

## Results

### Baseline Characteristics

64/287 patients (22.3%) had preformed DSA. DSA-positive patients were more likely to be female, more often underwent retransplantation and were more likely to receive a deceased-donor transplant compared to DSA-negative patients. The proportion of patients with elevated sCD30 levels was comparable between DSA-positive (39.1%) and DSA-negative (38.1%) patients (Supplementary Table 1). We next categorized patients according to pre-transplant DSA- and sCD30-status (DSA−/sCD30−, DSA−/sCD30+, DSA+/sCD30−, DSA+/sCD30+, Table 1). In DSA-positive patients, median MFI of the DSA with the highest MFI (MFI^max^) was comparable between sCD30-positive (5,528, range 1,129–20,379) and sCD30-negative (5,168, 1,051–21,994) patients. Also, sCD30 concentrations were comparable in the two sCD30-positive groups. Median follow-up was 7.4 years (range 0–15.7) with no significant differences between the groups (Table 1).

**Table 1:** Baseline characteristics (*n* = 287).

	**DSA−/sCD30−** **(*n* = 138)**	**DSA+/sCD30−** **(*n* = 39)**	**DSA−/sCD30+** **(*n* = 85)**	**DSA+/sCD30+(*n* = 25)**	***p*** **-value**
**Donor**					
Age, median (range)	57 (3–79)	56 (22–81)	56 (17–82)	52 (17–70)	0.580^#^
Female sex, *n* (%)	78 (56.5)	17 (43.6)	43 (50.6)	10 (40.0)	0.293^§^
Deceased donors, *n* (%)	78 (56.5)	29 (74.4)	42 (49.4)	19 (76.0)	0.016^§^
**Recipient**					
Age, median (range)	54 (19–74)	55,5 (31–73)	50 (17–78)	54 (18–73)	0.082^#^
Female sex, *n* (%)	34 (24.6)	17 (43.6)	33 (38.8)	11 (44.0)	0.029^§^
>1 KTX, *n* (%)	10 (7.2)	18 (46.2)	8 (9.4)	14 (56.0)	<0.001^§^
CDC-PRA <5, 5–84, >85 (%)					
Current	92.8/7.2/0	41/59/0	95.2/4.8/0	54.2/41.6/4.2	<0.001^§^
Highest	86.2/12.3/1.4	30.8/66.6/2.6	88.1/11.9/0	54.2/37.5/8.3	<0.001^§^
Number of HLA mismatches (A, B, DR), *n* (%)					0.177^§^
0	17 (12.3)	2 (5.1)	12 (14.1)	0 (0)	
1–2	35 (25.4)	8 (20.5)	22 (25.9)	10 (40.0)	
3–4	61 (44.2)	18 (46.2)	34 (40)	14 (56.0)	
5–6	25 (18.1)	11 (28.2)	17 (20)	1 (4.0)	
MFI^max^ median (range)	-	5,168 (1,051–21,994)	-	5,528 (1,129–20,379)	
sCD30 (U/ml), median (range)	-	-	99 (80–314)	100 (81–403)	
Follow-up (years) median (range)	7.6 (0–15.7)	8 (0–13.2)	6.8 (0–15.4)	6.85 (0.1–13.1)	0.298^#^

*KTX, kidney transplantation; CDC-PRA, complement-dependent cytotoxicity panel reactive antibodies; MFI^max^, mean fluorescence intensity value of the DSA with the highest MFI.*

*P-values were obtained by*

# *Kruskal-Wallis or*

§ *Chi-square testing. There were no statistically significant differences between the DSA+/sCD30− and the DSA+/sCD30+-group*.

### Allograft Survival

**Figure 1:**
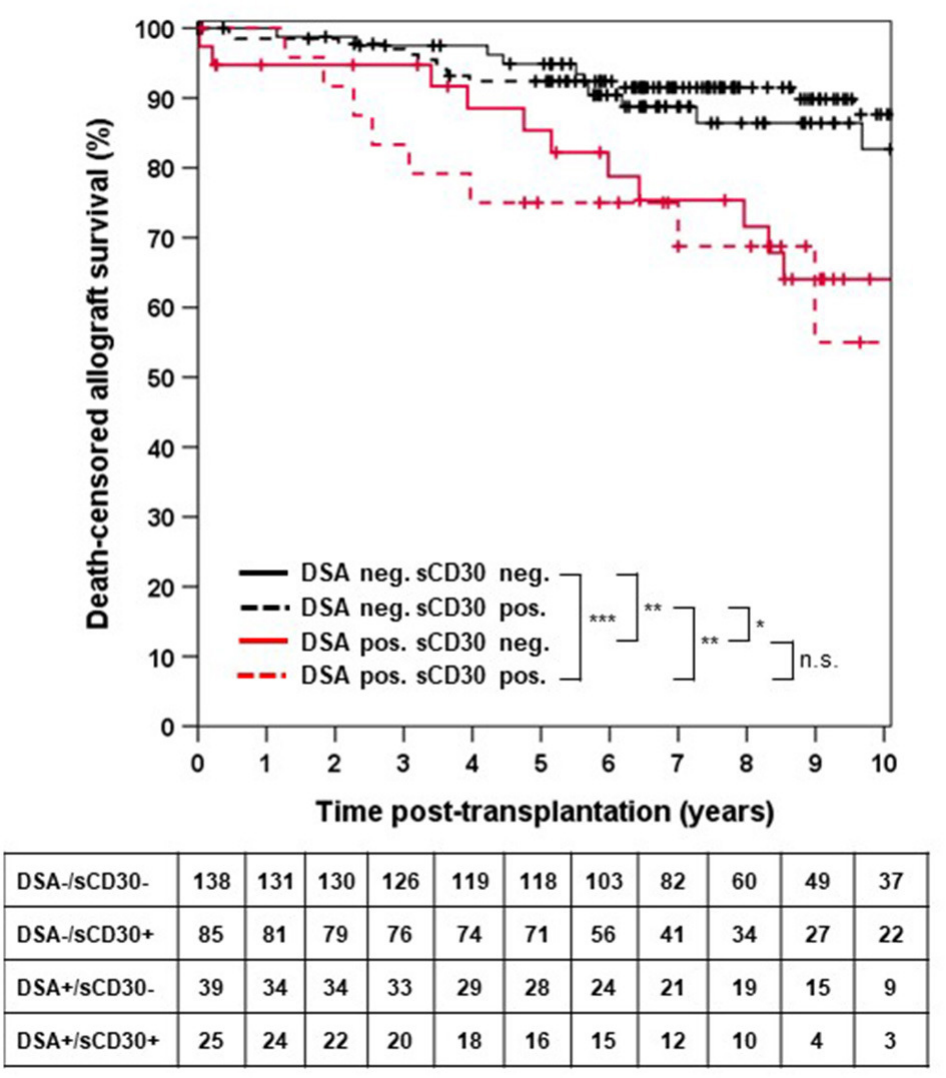
Death-censored allograft survival stratified by DSA and sCD30 status prior to transplantation. ***p < 0.001, **p = 0.001, *p = 0.014, and n.s. = non-significant.

**Figure 2:**
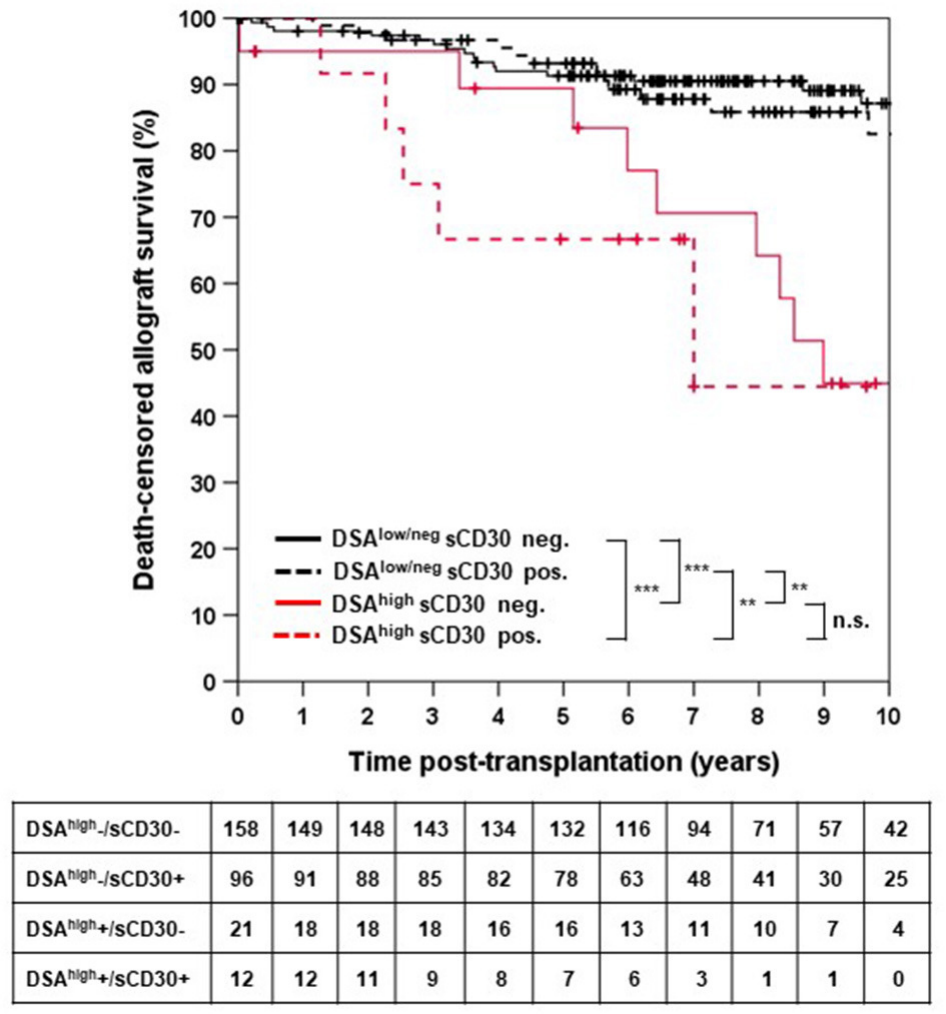
Death-censored allograft survival stratified by DSA^high^ and sCD30 status. DSA^high^ were defined as DSA with an MFI ≥ 5,000. DSA^low/neg^ indicates either no DSA or DSA with MFI between 500 and 5,000. ^***^p < 0.001, ^**^p < 0.01, and n.s. = non-significant.

Death-censored allograft survival was significantly lower in DSA-positive as compared to DSA-negative patients (10-year allograft survival 62.0 ± 7.3% vs. 85.9 ± 3.0%, *p* < 0.001; Supplementary Figure 1). When sCD30 was included into risk stratification, both sCD30-positive and sCD30-negative patients with preformed DSA had a significantly higher incidence of graft failure during follow-up as compared to DSA-negative patients (Figure 1). Of note, there was a trend toward accelerated graft failure in sCD30-positive as compared to sCD30-negative patients with preformed DSA (3-year allograft survival 83.3 ± 7.6 vs. 94.7 ± 3.6%, *p* = 0.177). Stratification of DSA-positivity by an MFI cutoff of 5,000 (DSA^high^) revealed that sCD30-positive DSA^high^ patients had the worst 3-year allograft survival (75.0 ± 12.5%), whereas sCD30-negative DSA^high^ patients had a 3-year allograft survival comparable to patients without DSA (95.0 ± 4.9 vs. 96.7 ± 1.4 and 96.7 ± 1.9%, respectively, Figure 2). Irrespective of the MFI cutoff applied (1,000 vs. 5,000), however, graft loss was only delayed in DSA-positive sCD30-negative patients, resulting in a significantly reduced allograft survival compared to the DSA-negative patient groups during follow up (10-year allograft survival 64.0 ± 8.9 vs. 82.7 ± 5.5 and 87.7 ± 3.6%, respectively, Figure 1).

### Incidence of Early ABMR

We hypothesized that the higher incidence of accelerated graft loss seen in sCD30-positive DSA-positive patients was due to a higher incidence of early rejection episodes, most notably ABMR. At the time of biopsy, however, a higher incidence of both T cell-mediated rejection (TCMR) and ABMR was noted in sCD30-negative as compared to sCD30-positive patients with preformed DSA within the first year (Table 2). As our study cohort comprised patients biopsied between 2005 and 2015 with a much higher awareness of ABMR reflected in the more recent Banff classifications, all kidney biopsies from DSA-positive patients were reevaluated and graded according to the Banff 2019 classification (20). This resulted in a much higher and comparable incidence of early ABMR in both groups (41.0 vs. 41.7%, Table 2). There was no statistically significant difference in the incidence of C4d-positive ABMR (37.5 vs. 20%, *p* = 0.41) or moderate microvascular injury (g+ptc ≥ 2) between the groups (75 vs. 100%, *p* = 1.00). Finally, we observed a trend toward more mixed rejections (50 vs. 20%, *p* = 0.218) and ABMR with v-lesions in sCD30-negative as compared to sCD30-positive patients (43.8 vs. 20%, *p* = 0.229).

**Table 2:** First year biopsy findings and treatment in DSA-positive patients.

	**DSA+/sCD30−** **(*n* = 39)**	**DSA+/sCD30+** (*n* = 24)^*^
Indication biopsy, *n* (% of all biopsies)	21 (53.8)	15 (62.5)
**Type of rejection at the time of biopsy**		
No rejection, *n* (%)	19 (48.7)	19 (80.0)
Borderline, *n* (%)	3 (7.7)	0 (0.0)
TCMR, *n* (%)	8 (20.5)	3 (12.0)
ABMR, *n* (%)	9 (23.1)	2 (8.0)
Received ABMR therapy, *n* (%)	7 (77.8)	1 (50.0)
**Reclassification according to Banff 2019**		
No rejection, *n* (%)	14 (35.9)	11 (45.8)
Borderline, *n* (%)	6 (15.4)	1 (4.2)
TCMR, *n* (%)	3 (7.7)	2 (8.3)
ABMR, *n* (%)	16 (41.0)	10 (41.7)
C4d-positive ABMR, *n* (%)	6 (37.5)	2 (20.0)
Combined rejection, *n* (%)	8 (50.0)	2 (20.0)
ABMR with v ≥ 1, *n* (%)	7 (43.8)	2 (20.0)
Time until ABMR (days) median (range)	8 (1–208)	7 (3–27)
Indication biopsy, *n* (% of biopsies with ABMR)	13 (81.3)	8 (80.0)
Received ABMR therapy, *n* (%)	7 (43.8)	3 (30.0)
Received ABMR therapy but lost graft, *n*/all graft losses following early ABMR (%)	5/10 (50.0)	3/7 (42.9)

* *1/25 patients did not receive a biopsy. There were no statistically significant differences between the groups*.

### Early Loss of DSA and Outcome

**Figure 3:**
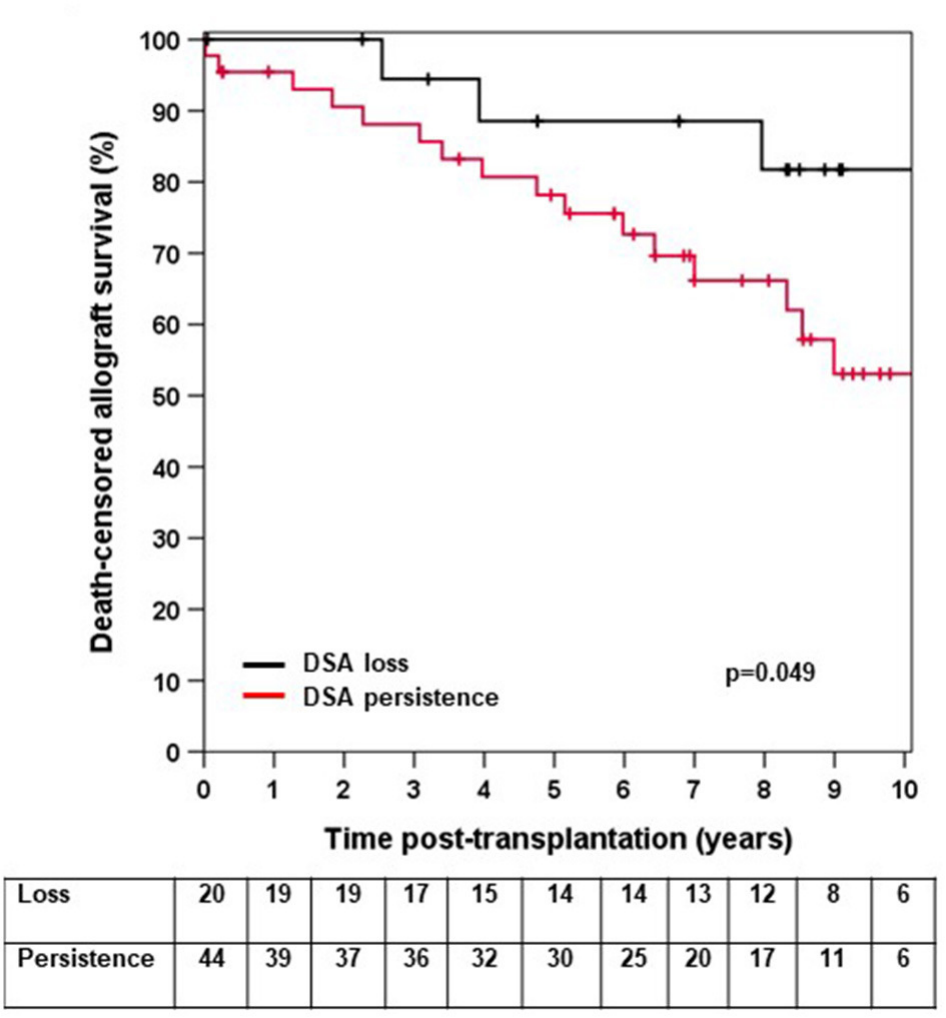
Death-censored allograft survival in DSA-positive patients in whom DSA were undetectable 14 days post-transplant (loss) or who had persistence of DSA (persistence).

**Figure 4:**
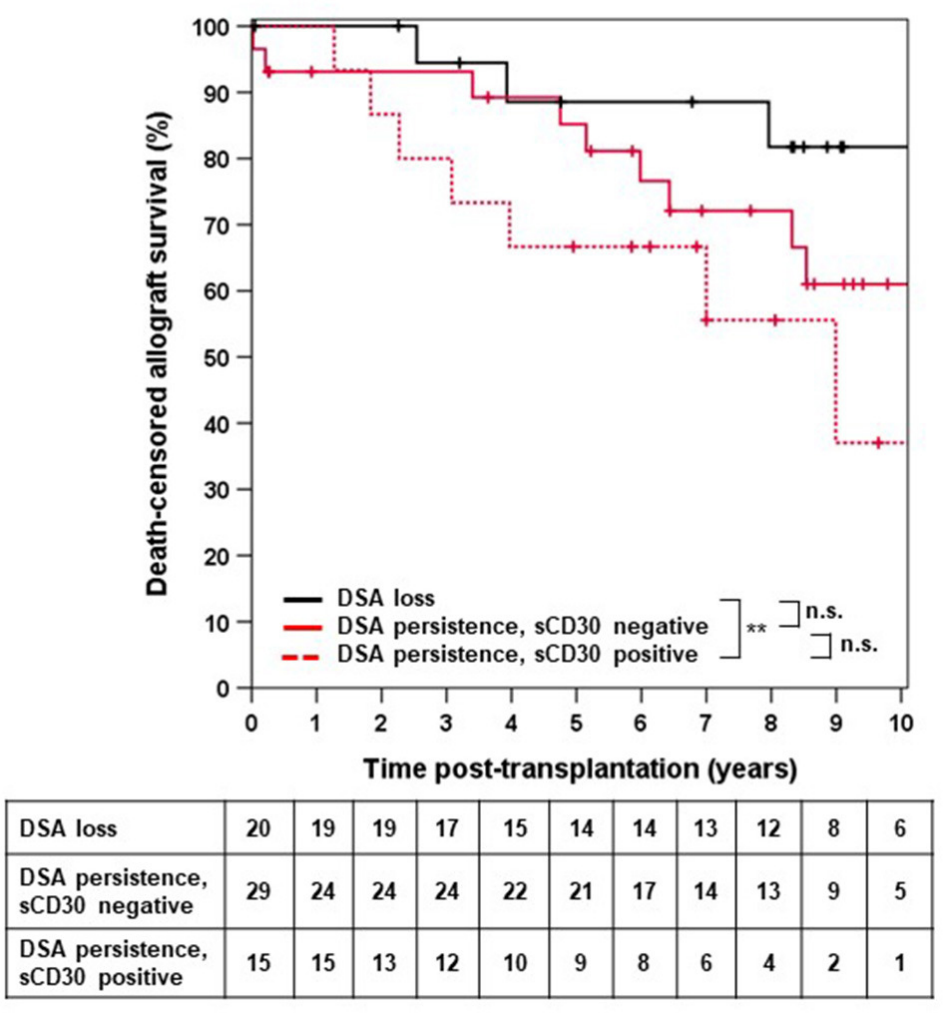
Death-censored allograft survival in DSA-positive patients in whom no DSA were detectable 14 days post-transplant irrespective of sCD30 status (loss) or who were either sCD30-positive (sCD30+) or sCD30-negative (sCD30^−^) prior to transplantation and had persistence of DSA 14 days post-KTX. **p = 0.008 and n.s. = non-significant.

We next asked whether elevated sCD30 levels as a surrogate marker of a preactivated immune system would be associated with a higher incidence of early DSA persistence. DSA-positive patients who lost their DSA as early as 14 days post-transplantation had very good outcomes, whereas persistence of DSA was associated with a significantly higher risk for impaired graft survival (10-year allograft survival 81.7 vs. 53.1%, *p* = 0.049, Figure 3). Of note, patients with persistent DSA had significantly higher MFI^max^ prior to transplantation compared to those patients in whom DSA were undetectable at day 14 (median 9,060 vs. 1,998, *p* < 0.001). However, the proportion of patients with persistent DSA 14 days post-transplantation was comparable between sCD30-positive (60%) and sCD30-negative (74.4%) patients (Table 3). Likewise, graft survival was not statistically different between these groups (Figure 4).

**Table 3:** DSA-status 14 days post-transplantation.

	**DSA+/sCD30–** **(*n* = 39)**	**DSA+/sCD30+** **(*n* = 25)**	***p*** **-value**
DSA persistence, *n* (%)	29 (74.4)	15 (60.0)	0.275^#^
DSA persistence in patients with ABMR, *n* (% of ABMR-pos. patients)	13 (81.3)	8 (100)	0.526^#^
Pre-transplant MFI^max^ of patients with DSA persistence, median (range)	7,478 (1,051–21,994)	10,178 (1,671–20,379)	0.240^§^
Pre-transplant MFI^max^ of patients with undetectable DSA at day 14, median (range)	1,664 (1,071–12,640)	2,037 (1,129–6,934)	0.395^§^

*P-values were obtained by*

# *chi-squared or*

§ *Mann-Whitney-U test*.

## Discussion

In this cohort of CDC-crossmatch negative patients with preformed DSA treated with a non-depleting induction regimen, both the incidence of early ABMR and long-term allograft survival were comparable in DSA-positive patients irrespective of the sCD30 status. Moreover, sCD30 status had no impact on the early dynamics of DSA post-transplantation.

In a previous study, Schaefer et al. described the incidence of ABMR and graft loss in a cohort of 80 highly sensitized patients with CDC-PRA > 85% that underwent desensitization prior to kidney transplantation (17). Detection of both DSA and high sCD30 levels prior to transplantation (*n* = 18) was associated with an increased risk for graft loss within 3 years following ABMR compared to the 43 DSA-positive but sCD30-negative patients (22 vs. 5%) (17). In our study, 2/16 (12.5%) sCD30-negative DSA-positive patients lost their graft within 3 years following ABMR as compared to 2/4 (50%) double-positive patients. Long-term follow-up of our study cohort, however, revealed a comparable incidence of graft loss following ABMR in both patient groups [7/10 (70%) in sCD30+DSA+ vs. 10/16 (62.5%) in sCD30-DSA+]. In our study, neither desensitization nor depleting induction treatment was performed. Consistent with other studies that re-evaluated histopathology specimens retrospectively (21), a considerable number of ABMR episodes had been missed at the time of biopsy (Table 2). Therefore, it is possible that in the absence of T cell help, reflected by low levels of sCD30, highly sensitized DSA-positive patients are particularly sensitive to both desensitization and/or rejection treatment.

In a second study on 385 sensitized (CDC-PRA- or anti-HLA ELISA-positive) patients that were transplanted between 1996 and 2011 without prior desensitization, Süsal et al. also found that pre-transplant DSA only carry an increased risk for graft loss within 5 years post-transplantation in the presence of high sCD30 levels. Data on the incidence of ABMR, however, were not reported (11, 18). 10-year follow-up revealed lower graft survival rates in all groups compared to our study even in the absence of both DSA and increased sCD30 levels (C. Süsal, data not shown), suggesting that the overall immunological risk was higher compared to our study population. Of note, all patients in the aforementioned studies received deceased-donor transplants. When we excluded living donor transplantations, we still did not observe significant differences in ABMR or outcome in DSA-positive patients with vs. without elevated sCD30 levels (Supplementary Figure 2). Likewise, when we analyzed overall graft loss not censored for death as reported in the study by Süsal et al. the differences between the groups remained unchanged (Supplementary Figure 3). Therefore, besides an increased immunological risk, differences in immunosuppression and post-transplant management between the two cohorts might explain some of the differences observed. Of note, our cohort and the one studied by Süsal et al. were not tested for the occurrence of de novo DSA or sCD30 levels post-transplantation. It would be interesting to find out whether increased levels of sCD30 either prior to or early after transplantation are predictive for the development of *de novo* DSA as an additional risk factor for a reduced allograft survival (22).

Our study confirms our previous results that disappearance of DSA as early as 14 days post-transplant is associated with very good outcomes (23). Most studies on DSA persistence reported data between 3 months (24) and 1 year (25, 26) post-transplant. Therefore, our data are very important, as early adaptation of immunosuppression and close monitoring of patients with DSA persistence might improve outcome.

Our study has several limitations. Besides the small number of patients and the inherent limitations of a single-center design, donor HLA typing was incomplete (as it has been for long in the Eurotransplant kidney allocation system) and could not be completed as donor DNA was not available to us. However, as outlined in the methods section, binary assignment of donor-specificity was not affected by the incomplete HLA typing. In addition, there were no predefined criteria for the use of basiliximab induction during the study period and CDC-PRA-positivity might have been a selection criterion in some cases. However, as the key advantage of the SAB assay is the ability to detect the specific sensitization against non-self HLA as compared to the CDC-PRA (27), stratification of our cohort by CDC-PRA and sCD30 status was not superior to a DSA-based approach (Supplementary Figure 4).

Our study has several strengths. First, both Luminex and ELISA analyses were performed retrospectively, so that risk stratification and treatment strategies were not influenced by DSA- and sCD30 status. Therefore, the prognostic value of these two biomarkers could be analyzed without the interference of desensitization or depleting induction therapy. Second, all analyses were performed from the same day of transplant serum and with the same reagents, reducing assay variability to a minimum. Third, follow-up was longer than in previous studies allowing for the detection of late allograft losses. Finally, meticulous re-analysis of histopathology according to the Banff 2019 classification revealed a comparable incidence of ABMR in DSA-positive patients irrespective of the sCD30 status, which further supports our outcome data.

In sum, in our cohort, preformed DSA were associated with an increased risk for ABMR and long-term graft loss independent of sCD30 levels in intermediate-risk kidney transplant patients. Therefore, determination of sCD30 in addition to SAB-determined DSA does not improve risk stratification prior to kidney transplantation.

## Data Availability Statement

The raw data supporting the conclusions of this article will be made available by the authors, without undue reservation.

## Ethics Statement

The studies involving human participants were reviewed and approved by Ethics Committee of Regensburg University. The patients/participants provided their written informed consent to participate in this study.

## Author Contributions

TD analyzed data and wrote the manuscript. CB, ML, and BS performed Luminex analyses and data interpretation. KU and MB-H performed histological analyses of kidney biopsies. BB gave conceptual advice and contributed to the writing of the manuscript. DZ designed the study, analyzed data, and wrote the manuscript. All authors contributed to the article and approved the submitted version.

## Conflict of Interest

The authors declare that the research was conducted in the absence of any commercial or financial relationships that could be construed as a potential conflict of interest.

## Publisher's Note

All claims expressed in this article are solely those of the authors and do not necessarily represent those of their affiliated organizations, or those of the publisher, the editors and the reviewers. Any product that may be evaluated in this article, or claim that may be made by its manufacturer, is not guaranteed or endorsed by the publisher.

## Abbreviations

AM: acceptable mismatch
ABMR: antibody-mediated rejection
CDC: complement-dependent cytotoxicity
CM: crossmatch
DGI: Deutsche Gesellschaft für Immungenetik
DSA: donor-specific antibodies
ET: Eurotransplant
ETKAS: Eurotransplant kidney allocation system
ESP: Eurotransplant senior program
FCM: flow cytometry crossmatch
GFR: glomerular filtration rate
HLA: human leukocyte antigen
IVIG: intravenous immunoglobulin
MFI: mean fluorescence intensity
PRA: panel-reactive antibodies
SAB: single antigen bead
sCD30: soluble CD30
TNF: tumor necrosis factor
UAM: unacceptable antigen mismatches
vPRA: virtual PRA.
