# Supplementary material for: Increased Levels of sCD30 Have No Impact on the Incidence of Early ABMR and Long-Term Outcome in Intermediate-Risk Renal Transplant Patients With Preformed DSA
Source: Front Med (Lausanne). 2021 Nov 8;8:778864. doi: 10.3389/fmed.2021.778864 (PMC8606593; doi:10.3389/fmed.2021.778864)
Supplement: Supplementary file 1 [file Data_Sheet_1.docx]

**Supplementary Digital Content**

| **Table S1. Baseline characteristics of study cohort** | | |
| --- | --- | --- |
|  | **DSA negative (n=223)** | **DSA positive**  **(n=64)** |
| **Recipient** |  |  |
| Age, median (range) | 52 (17-78) | 55 (18-73) |
| Female sex, n (%) | 67 (30) | 28 (43,8) |
| >1 KTX, n (%) | 18 (8,1) | 32(50) |
| **Donor** |  |  |
| Age, median (range) | 56 (3-82) | 53,5 (17-81) |
| Female sex, n (%) | 121 (54,3) | 27 (42,2) |
| Deceased donors,  n (%) | 120 (53,8) | 48 (75) |
| CDC-PRA <5, 5-84, >85 (%) |  |  |
| Current | 93.7/6.4/0 | 46/52.4/1.6 |
| Highest | 86.9/12.2/0.9 | 39.7/55.5/4.8 |
| Cumulative number of HLA mismatches  (A, B, DR), n (%) |  |  |
| 0 | 29 (13.0) | 2 (3.2) |
| 1-2 | 57 (25.5) | 17 (27.0) |
| 3-4 | 95 (42.6) | 32 (50.8) |
| 5-6 | 42 (18.8) | 12 (19.0) |
| MFI^max^, median (range) | - | 5348  (1051 - 21994) |
| sCD30-positive, n (%) | 85 (38.1) | 25 (39.1) |
| Basiliximab-Induction, % | 100 | 100 |
| CyA-MMF-Pred, % | 3.1 | 4.7 |
| FK-MMF-Pred, % | 96.9 | 95.3 |
| Follow up, median (range) (years) | 7.4 (0 - 15.7) | 7 (0 - 13.2) |
| KTX, kidney transplantation, CDC-PRA, complement-dependent cytotoxicity panel reactive antibodies; MFI^max^, mean fluorescence intensity of the DSA with the highest MFI; CyA, Cyclosporine A, FK, Tacrolimus. | | |

**Figure S1**


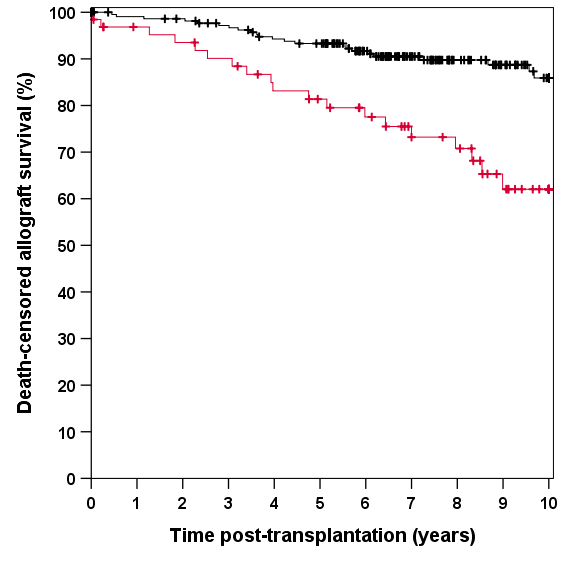


**p<0.001**

**DSA-negative**

**DSA-positive**

**
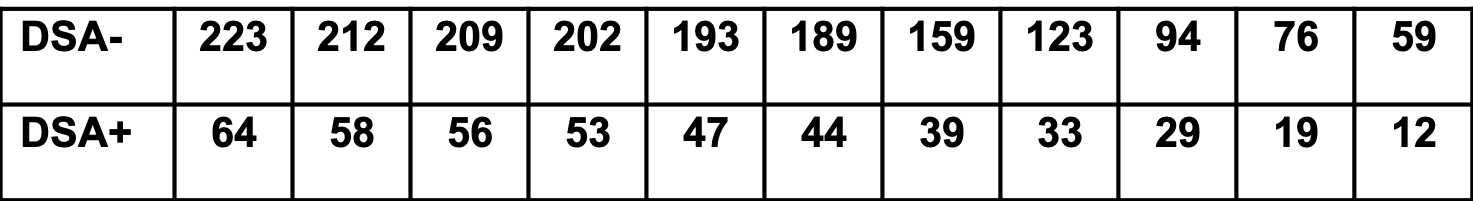
**

Figure S1. Death-censored allograft survival in patients with (DSA-positive) and patients without DSA (DSA-negative).

**Figure S2**


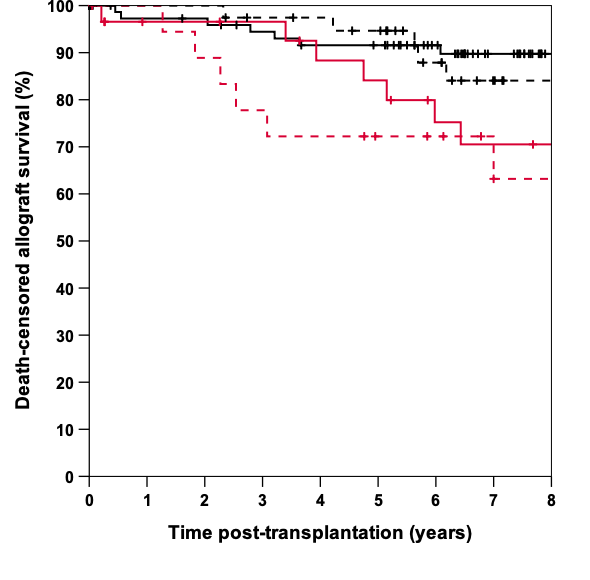


**DSA neg. sCD30 neg.**

**DSA neg. sCD30 pos.**

**DSA pos. sCD30 neg.**

**DSA pos. sCD30 pos.**

***

**

**

*

n.s.

| **DSA-/sCD30-** | **78** | **71** | **70** | **66** | **63** | **62** | **51** | **36** | **22** |
| --- | --- | --- | --- | --- | --- | --- | --- | --- | --- |
| **DSA-/sCD30+** | **42** | **39** | **39** | **36** | **35** | **33** | **25** | **18** | **15** |
| **DSA+/sCD30-** | **29** | **25** | **25** | **24** | **21** | **20** | **16** | **15** | **14** |
| **DSA+/sCD30+** | **19** | **18** | **16** | **14** | **13** | **11** | **10** | **8** | **6** |

Figure S2. Death-censored allograft survival in recipients of deceased-donor transplants (n=168) stratified by DSA and sCD30 status prior to transplantation. ***, p< 0.001; **, p=0.002; *, p=0.035; n.s., non-significant.

**Figure S3**


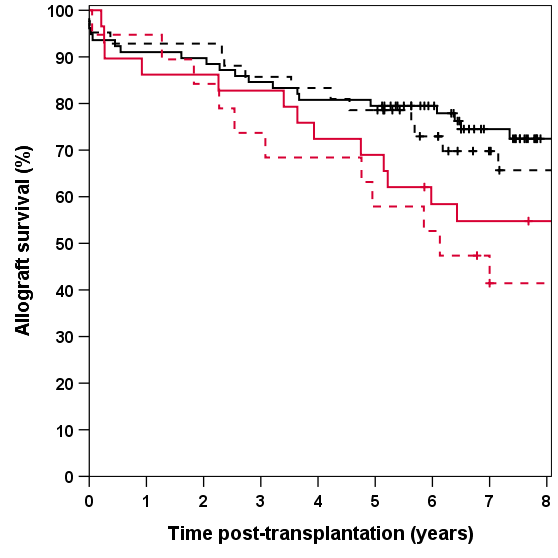


| **DSA-/sCD30-** | **78** | **71** | **70** | **66** | **63** | **62** | **51** | **36** | **22** |
| --- | --- | --- | --- | --- | --- | --- | --- | --- | --- |
| **DSA-/sCD30+** | **42** | **39** | **39** | **36** | **35** | **33** | **25** | **18** | **15** |
| **DSA+/sCD30-** | **29** | **25** | **25** | **24** | **21** | **20** | **16** | **15** | **14** |
| **DSA+/sCD30+** | **19** | **18** | **16** | **14** | **13** | **11** | **10** | **8** | **6** |

**DSA neg. sCD30 neg.**

**DSA neg. sCD30 pos.**

**DSA pos. sCD30 neg.**

**DSA pos. sCD30 pos.**

***

**

*

n.s.

n.s.

Figure S3. Allograft survival including non-immunological causes (incl. primary non-function with a biopsy excluding rejection and recurrent primary disease) and not censored for death in recipients of a deceased-donor transplant (n=168) stratified by DSA and sCD30 status prior to transplantation. ***, p< 0.001; **, p=0.004; *, p=0.018; n.s., non-significant.

Figure S4


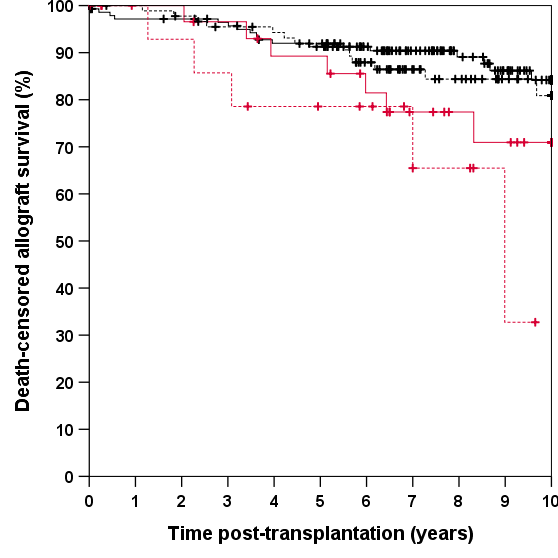


**CDC-PRA neg. sCD30 neg.**

**CDC-PRA neg. sCD30 pos.**

**CDC-PRA pos. sCD30 neg.**

**CDC-PRA pos. sCD30 pos.**

**

*

*

n.s.

n.s.

| **PRA-/sCD30-** | **144** | **136** | **135** | **132** | **124** | **122** | **107** | **88** | **67** | **53** | **38** |
| --- | --- | --- | --- | --- | --- | --- | --- | --- | --- | --- | --- |
| **PRA-/sCD30+** | **93** | **89** | **86** | **82** | **80** | **76** | **61** | **45** | **38** | **28** | **23** |
| **PRA+/sCD30-** | **33** | **29** | **29** | **27** | **24** | **24** | **20** | **15** | **12** | **11** | **8** |
| **PRA+/sCD30+** | **15** | **14** | **13** | **12** | **10** | **9** | **8** | **6** | **4** | **2** | **0** |

Figure S4. Death-censored allograft survival stratified by CDC-PRA >5% (CDC-PRA positive) and sCD30 status prior to transplantation. **, p< 0.01; *, p<0.05; n.s., non-significant.
